# The association between brominated flame retardants exposure with Parkinson’s disease in US adults: a cross-sectional study of the National Health and Nutrition Examination Survey 2009–2016

**DOI:** 10.3389/fpubh.2024.1451686

**Published:** 2024-10-21

**Authors:** Jia-jie Lv, Yi-chi Zhang, Xin-yu Li, Lin-jie Zhang, Zhuo-ma Yixi, Cheng-hao Yang, Xu-hui Wang

**Affiliations:** ^1^Department of Vascular Surgery, Shanghai Putuo People’s Hospital, School of Medicine, Tongji University, Shanghai, China; ^2^Department of Vascular Surgery, Shanghai Ninth People’s Hospital, Shanghai Jiao Tong University, Shanghai, China; ^3^Department of Plastic and Reconstructive Surgery, Shanghai Ninth People’s Hospital, Shanghai Jiao Tong University School of Medicine, Shanghai, China

**Keywords:** brominated flame retardants, Parkinson’s disease, The National Health and Nutrition Examination Survey, cross-sectional study, BKMR analysis

## Abstract

**Background:**

Increasing evidence suggests that environmental factors play a crucial role in the pathogenesis of Parkinson’s disease (PD). Humans are simultaneously exposed to multiple brominated flame retardants (BFRs) in the environment. However, the relationship between BFRs and PD remains unclear. This study was designed to investigate the overall association between BFRs and PD in a nationally representative US population and to further identify significant chemicals.

**Methods:**

This study used data from 7,161 NHANES participants from 2009 through 2016. The serum BFRs registry included PBDE-28, PBDE-47, PBDE-85, PBDE-99, PBDE-100, PBDE-153, PBDE-154, PBDE-183, PBDE-209, and PBB-153. A survey-weighted generalized logistic regression model with restricted cubic splines (RCS) was used to evaluate the association between single BFRs exposure and periodontitis. Meanwhile, weighted quantile sum (WQS) regression and Bayesian kernel machine regression (BKMR) were used to evaluate the overall association of mixed frankincense powder with periodontitis and to identify significant chemicals. Sensitivity analysis was performed to evaluate the robustness of the results.

**Results:**

Among the 7,161 participants, 65 had PD. PD patients were older (mean age 57.79 vs. 46.57 years) and had a higher proportion of females (70.86%) compared to non-PD participants. Serum levels of PBB-153 were significantly higher in those with PD. Logistic regression analyses revealed a non-linear, inverted U-shaped relationship between serum PBB-153 and PD risk. The risk of PD increased with higher PBB-153 levels up to the 3rd quartile (Q3), beyond which the risk declined (Q3 vs. Q1: OR = 4.98, 95% CI = 1.79–13.86; Q4 vs. Q1: OR = 3.23, 95% CI = 1.03–10.08). PBB-153 (43.40%), PBDE-153 (24.75%), and PBDE-85 (19.51%) contributed most to the weighted quantile sum index associated with PD risk. Bayesian kernel machine regression confirmed the inverted U-shaped dose–response pattern for PBB-153 and the overall BFR mixture. Restricted cubic spline analyses corroborated the non-linear relationship between PBB-153 and PD, which was more pronounced among women and those aged 37–58 years. Sensitivity analyses substantiated these findings.

**Conclusion:**

This nationally representative cross-sectional study revealed a novel non-linear, inverted U-shaped relationship between serum levels of the brominated flame retardant PBB-153 and Parkinson’s disease risk in U.S. adults. The risk increased with higher PBB-153 exposure up to a point, beyond which it declined. This complex dose–response pattern highlights the importance of considering potential hormetic mechanisms and effect modifiers when evaluating environmental exposures and neurodegenerative diseases. Further research is warranted to elucidate the underlying biological pathways and inform risk mitigation strategies.

## Introduction

Parkinson’s disease (PD) is a progressive neurodegenerative disorder distinguished by motor symptoms such as tremors, rigidity, bradykinesia, and postural instability, alongside non-motor manifestations including cognitive impairment, autonomic dysfunction, and sleep disturbances (1). It is the second most prevalent neurodegenerative disease after Alzheimer’s disease, affecting approximately 1% of individuals over the age of 60 (2). Despite substantial research efforts, the precise etiology of PD remains largely unresolved, with both genetic and environmental factors implicated in its pathogenesis.

Brominated flame retardants (BFRs) are a group of synthetic compounds extensively employed in consumer products such as electronics, furniture, and textiles to reduce flammability (3). Concerns have been raised about these compounds due to their environmental persistence, bioaccumulative potential, and possible adverse health effects. A number of studies have examined the association between BFR exposure and various health outcomes, including neurodevelopmental disorders, endocrine disruption, and carcinogenesis (4, 5).

The potential link between BFR exposure and neurodegenerative diseases like PD has been explored, though the findings are not definitive. Certain studies suggest that specific BFRs, notably polybrominated diphenyl ethers (PBDEs), may contribute to PD pathogenesis by mechanisms such as oxidative stress, neuroinflammation, and mitochondrial dysfunction (6, 7). Conversely, other investigations have reported no significant association or have presented conflicting evidence (8, 9).

Given the potential implications of BFR exposure for PD risk, the current body of evidence remains limited and necessitates further exploration. Most existing studies have concentrated on specific BFR congeners or have been conducted in animal models or *in vitro* systems, underscoring the need for comprehensive evaluations of BFR exposure and its possible connection to PD in human populations.

These inconsistent findings underscore the critical need for additional research to clarify the potential relationship between BFR exposure and PD, particularly within the context of the U.S. population, where BFR usage is widespread. Understanding this association has significant implications for public health, environmental policy, and the development of preventive and therapeutic strategies for age-related diseases, including Parkinson’s disease. To address this gap in knowledge, we conducted a secondary analysis of data from the National Health and Nutrition Examination Survey (NHANES), controlling for multiple confounders. We employed various models to investigate the association between individual and combined BFR exposure and the incidence of PD in U.S. adults.

## Materials and methods

### Study population

**Figure 1:**
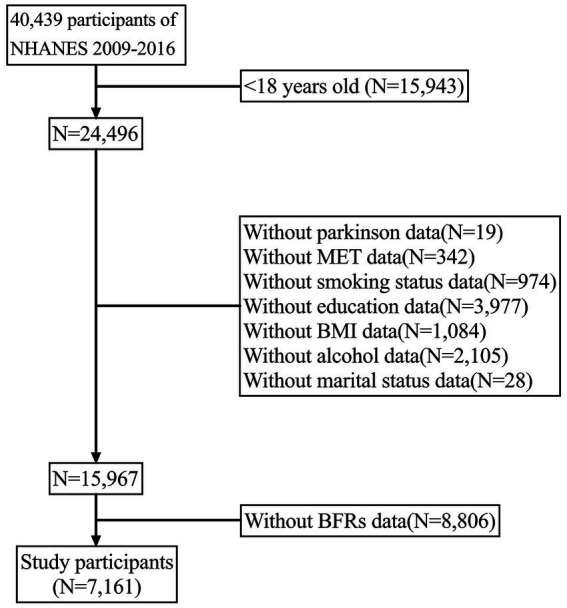
Flow chart of the study.

The National Health and Nutrition Examination Survey (NHANES) is a broad interdisciplinary research project initiated by the Centers for Disease Control and Prevention (CDC) to assess the health and nutritional status of populations in the United States. The primary goal of NHANES is to collect, analyze, and publish health, nutrition, and environmental exposure data in the United States. It has been conducted annually since the 1960s and covers all age groups in the country. In this study, PD data were investigated and four survey periods (2009–2010, 2011–2012, 2013–2014, and 2015–2016) were combined to improve accuracy. The study included 7,161 participants aged between 18 and 80 years. Figure 1 outlines the inclusion and exclusion criteria for this study. The NHANES study ethics review board approved the study protocol for the period 2009–2016, and all participants provided written informed consent.

### Exposure: brominated flame retardants

In the NHANES dataset, PBB-153 and 9 PBDEs in serum were measured using automated liquid–liquid extraction and subsequent sample purification (NHANES, 2019). However, in this study, we only considered PBB-153 and nine PBDEs with a detection rate greater than 50% (10). Namely, 2,4,4′-tribromodiphenyl ether (BDE-28), 2,2′,4,4′- tetrabromodiphenyl ether (BDE-47), 2,2′,3,4,4′-pentabromodiphenyl ether (BDE-85), 2,2′,4,4′,5-pentabromodiphenyl ether (BDE-99), 2,2′,4,4′,6-pentabromodiphenyl ether (BDE-100), 2,2′,4,4′,5,5′- hexabromodiphenyl ether (BDE-153), 2,2′,4,4′,5,6′-hexabromodiphenyl ether (BDE-154), 2,2′,3,4,4′,5′,6-heptabromodiphenyl ether (BDE-183), decabromodiphenyl ether (BDE-209), and 2,2′,4,4′,5, 5′-hexabromobiphenyl (PBB-153).

### Outcome: Parkinson’s disease

In this study, participants were identified with PD by specifying “Second Level Category Name” as “ANTIPARKINSON AGENTS” in the Prescription Medications document. Based on the responses to questions regarding prescription medication, this determination was made. Since this method of identification was limited by medications and codes included in the NHANES, an individual had to be receiving treatment for PD to be classified as having it. Those who did not report taking an anti-parkinsonian medication were classified as not having PD. The definition of the disease is based on previous studies (11, 12).

### Covariates

This study considered several sociodemographic characteristics based on previous research, including age, sex, race, education level, marital status, poverty income ratio (PIR), smoking status, drinking status, body mass index (BMI), metabolic equivalent (MET), and comorbidity index (CCI). Race was categorized as Mexican American, white, black, or other. Education level was less than high school, high school, or college or above. Marital status was living with a peer, single, or married. PIR measured socioeconomic status as the ratio of household income to poverty line, categorized as low, medium, or high. Smoking status was coded as never, former, or current smoker. Drinking status included never, former, mild, moderate, and heavy drinker categories. Comorbidities included conditions like diabetes, heart disease, lung disease, hypertension, and cancer. A CCI score calculated the number and severity of conditions, with higher scores indicating greater impact on prognosis.

### Statistical analysis

Chi-square test and t-test were used to evaluate the demographic characteristics of subjects with different PD states. Serum BFRs were Ln transformed to obtain an approximately normal distribution (continuous variables) or divided into four quartiles (Q1, Q2, Q3, and Q4) as categorical variables. Multivariate logistic regression model was used to analyze the association between serum BFRs and the risk of PD. All analyses were performed for age, sex (male or female), race/ethnicity (Mexican American, other Hispanic, non-Hispanic white, non-Hispanic black, or other), education, marital status, metabolic equivalent (MET), alcohol use status, body-mass index (BMI), household income/poverty ratio (PIR), and other covariates Correction. Pearson correlation analysis was used to assess the correlation of Ln conversion to BFRs. Because the weighted quantile sum (WQS) regression performed well in describing environmental mixtures, we used this regression to explore the overall effect of serum BFRs on PD onset. The R package (“gWQS “) allows empirical calculation of a WQS index consisting of a weighted sum of individual BFRs concentrations. The WQS index ranges from 0 to 1, representing the level of mixed exposure to brominated furoic acid, and components of concern with non-negligible weights were identified. The final results were interpreted as the simultaneous effect of adding a quantile of mixed BFRs on PD. In addition, we conducted a Pearson correlation analysis to examine the relationships between different PBDE congeners. This analysis helps to understand whether the PBDEs are interrelated, which could influence their cumulative effects on health outcomes (Supplementary Figure 1).

First, weighted multiple logistic regression analysis was used to evaluate the association between serum BFRs and PD. Finally, three different models were constructed. No adjustments were made to the original model. Model 1 was adjusted for age, sex, and race; Based on Model 1, Model 2 was further adjusted for other demographic factors (race, BMI, PIR, MET, and education), CCI, and lifestyle factors (such as smoking and alcohol consumption). Second, considering the potential nonlinear, non-additive relationship between bromamphetamine and PD, Bayesian kernel machine regression (BKMR) was used to assess the combined effect of all bromamphetamine and PD prevalence (“BKMR “package) and the dose–response relationship between single bromamphetamine and PD prevalence to determine the risk of other bromamphetamine and PD prevalence as a function of concentration. 5,000 simulation analyses based on normal approximation were performed using the quasi-Bayesian Monte Carlo method. Subsequently, after screening, we found that PBB-153 was significantly associated with PD incidence. In order to clarify the nonlinear relationship between the two, we performed smooth curve fitting (penalty spline method). Finally, to assess the robustness of the results, we performed sensitivity analyses targeting people younger than 60 years to further validate the association between PBB-153 and PD onset. Statistical analyses were performed with the use of R software 4.3.0 (Core Team, Vienna, Austria). *p* < 0.05 was considered statistically significant.

## Results

### Characteristics of participants

A total of 7,161 subjects were included in the study, including 65 patients with Parkinson’s disease. Table 1 lists the characteristics of the participants according to PD and non-PD groups. The average age of PD patients was 57.79 years, which was significantly higher than 46.57 years of non-PD patients (*p* < 0.0001). Among the patients, the proportion of female patients was significantly higher than that of male patients (70.86% vs. 29.14%). Participants’ age, sex, race, marital status, MET, and CCI were also significantly different by PD status (all *p* < 0.05, Table 1). In addition, education, household income, alcohol consumption, and smoking status were also not statistically different between PD and non-PD patients. Furthermore, in response to serum BFR concentrations, serum PBB155 was significantly higher in participants with PD than in those without PD (Table 1).

**Table 1:** Characteristics of participants by Parkinson’s disease, NHANES 2009–2016.

Variable	Total	Non-Parkinson	Parkinson	*p*
**Age (years, SD)**	46.68 (0.40)	46.57 (0.40)	57.79 (2.11)	<0.0001
**Gender (*n*, %)**				0.02
Female	3,716 (51.89)	3,674 (52.16)	42 (70.86)	
Male	3,445 (48.11)	3,422 (47.84)	23 (29.14)	
**Race/ethnicity (*n*, %)**				0.003
Mexican American	1,106 (15.44)	1,097 (8.73)	9 (6.98)	
Non-Hispanic Black	1,538 (21.48)	1,528 (11.56)	10 (7.86)	
Non-Hispanic White	2,805 (39.17)	2,765 (65.40)	40 (82.00)	
Other Hispanic	768 (10.72)	765 (5.97)	3 (1.09)	
Other race/ethnicity	944 (13.18)	941 (8.33)	3 (2.07)	
**Marital status (*n*, %)**				0.005
Married/cohabiting	4,377 (61.12)	4,347 (64.81)	30 (51.88)	
Never married	1,274 (17.79)	1,262 (17.16)	12 (11.37)	
Widowed/divorced/separated	1,510 (21.09)	1,487 (18.04)	23 (36.75)	
**Education (*n*, %)**				0.49
Above high school	3,731 (52.1)	3,699 (61.42)	32 (60.35)	
High school or equivalent	1,615 (22.55)	1,604 (21.93)	11 (17.30)	
Under high school	1,815 (25.35)	1,793 (16.65)	22 (22.34)	
**PIR (SD)**				0.39
[0, 1.3)	2,218 (34.23)	2,195 (22.73)	23 (26.71)	
[1.3, 3.5]	2,354 (36.33)	2,332 (35.46)	22 (41.57)	
(3.5, 5]	1,907 (29.43)	1,897 (41.81)	10 (31.73)	
**BMI (kg/m** ^**2**^ **, %)**				0.16
[14.1, 18.5)	129 (1.82)	128 (1.90)	1 (0.58)	
[18.5, 28]	3,484 (49.24)	3,463 (50.14)	21 (38.02)	
(28, 81.3]	3,462 (48.93)	3,422 (47.95)	40 (61.40)	
**MET (SD)**	4,601.83 (133.33)	4,623.03 (133.32)	2,191.45 (674.44)	<0.001
**Alcohol consumption (*n*, %)**				0.74
Heavy	1,052 (14.69)	1,038 (12.98)	14 (10.69)	
Mild	1,331 (18.59)	1,324 (19.85)	7 (21.93)	
Moderate	2,811 (39.25)	2,779 (40.41)	32 (47.75)	
Former	936 (13.07)	932 (16.29)	4 (12.23)	
Never	1,031 (14.4)	1,023 (10.46)	8 (7.39)	
**Smoking status (*n*, %)**				0.25
Former	1,599 (22.9)	1,580 (23.56)	19 (29.99)	
Never	4,013 (57.48)	3,981 (56.74)	32 (43.98)	
Now	1,370 (19.62)	1,356 (19.71)	14 (26.04)	
**CCI (SD)**	0.97 (0.02)	0.96 (0.02)	2.15 (0.43)	0.01
**Brominated flame retardants (SD)**				
PBDE28	0.00 (0.01)	0.00 (0.01)	−0.05 (0.09)	0.57
PBDE47	2.89 (0.01)	2.89 (0.01)	2.76 (0.11)	0.25
PBDE85	−1.02 (0.01)	−1.02 (0.01)	−1.14 (0.09)	0.15
PBDE99	1.24 (0.01)	1.24 (0.01)	1.10 (0.12)	0.28
PBDE100	1.32 (0.01)	1.32 (0.01)	1.17 (0.10)	0.14
PBDE153	2.30 (0.01)	2.30 (0.01)	2.40 (0.12)	0.41
PBDE154	−1.12 (0.01)	−1.12 (0.01)	−1.28 (0.10)	0.11
PBDE183	−1.53 (0.01)	−1.53 (0.01)	−1.65 (0.07)	0.08
PBDE209	0.86 (0.01)	0.87 (0.01)	0.71 (0.08)	0.05
PBB153	0.79 (0.03)	0.78 (0.03)	1.12 (0.11)	0.01

SD, Standard Deviation. PIR, Poverty to income ratio; BMI, Body mass index; CCI, Charlson comorbidity index; BFRs, Brominated flame retardants.

### Associations between serum BFRs and PD risk

**Figure 2:**
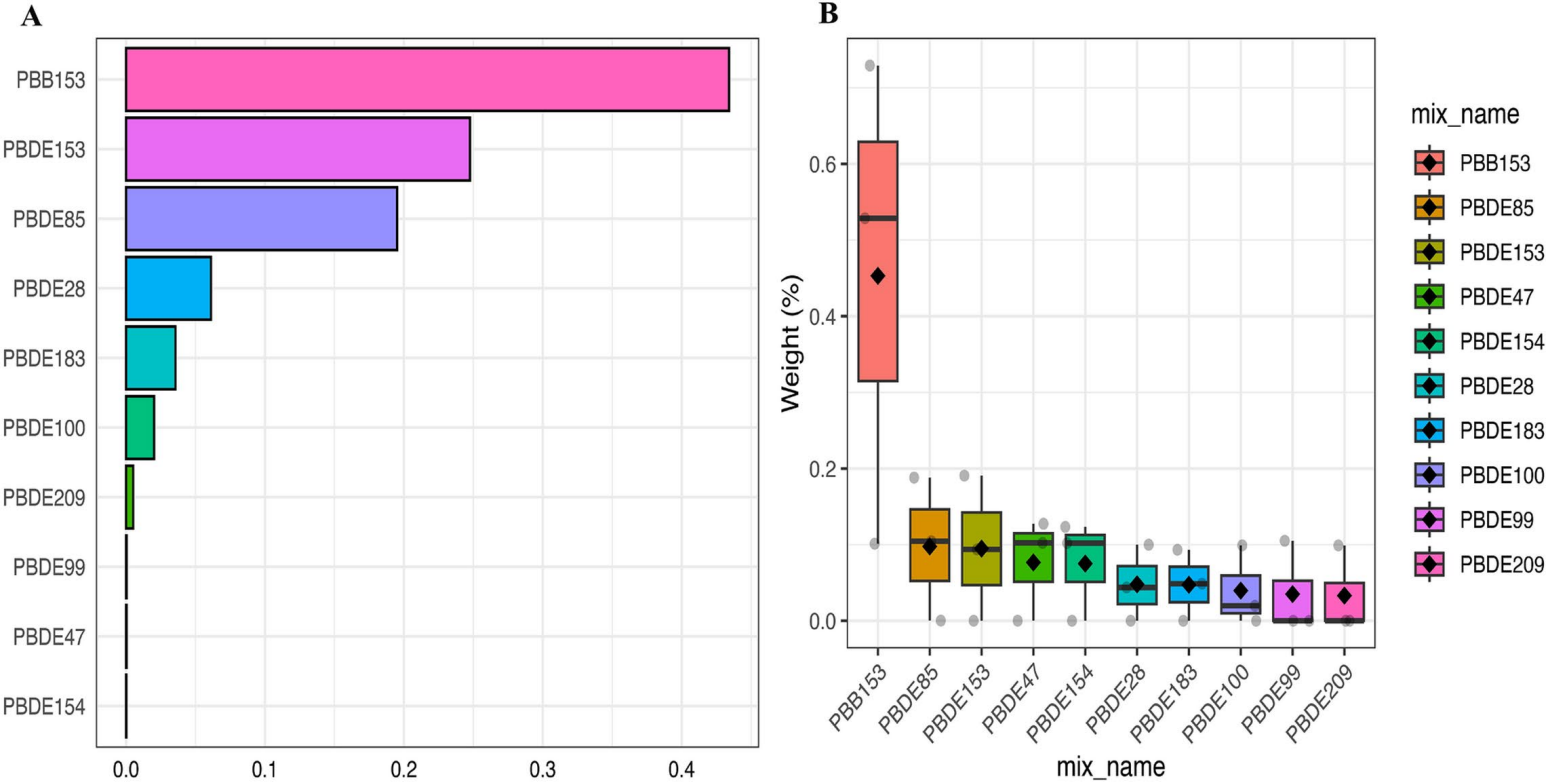
Weighted values of brominated flame retardants for PD in WQS models. **(A)** Bar Chart Representation of Top Compounds, **(B)** Box Plot of Weight Distribution Across Compounds. Models were adjusted for gender, age, race, education, PIR, marital status, BMI, MET, drinking alcohol status, smoking status and CCI.

In the multiple Logistic regression analysis, each serum BFRs concentration was Ln transformed to fit a normal distribution. After controlling for confounding factors, there was a significant positive correlation between PBB-153 PD and 8 kinds of BFRs (*p* < 0.05). In the original model, with each unit increase of PBB-153 concentration, the prevalence of PD increased by 32% (OR: 1.32, 95%CI: 1.09, 1.61, *p* < 0.05). In model 1 (adjusted for gender and race), each unit increase in PBB153 concentration was associated with 31% increased prevalence of PD (OR: 1.31, 95%CI: 1.05, 1.64, *p* < 0.05). In model 2 (adjusted for related factors based on model 1), each unit increase in PBB153 concentration was associated with a 23% increase in PD prevalence (OR: 1.23, 95%CI: 0.96, 1.57, *p* < 0.05). Other BFRs were positively or negatively correlated with PD incidence, but the results were not statistically significant. Secondly, BFRs were divided into quartiles (Q1–Q4) to explore the relationship between different concentrations of BFRs and the risk of PD. After controlling for potential confounders, a significant dose–response relationship was found between PBB153 and the risk of PD (*p* for trend <0.001, Table 2). The risk of PBB-153 Q3 was 4.98 times that of Q1 (OR = 4.98, 95%CI: 1.79, 13.86, *p* < 0.05). The risk of PBB-153Q4 was 3.23 times higher than that of Q1 (OR = 3.23, 95%CI: 1.03, 10.08, *p* < 0.05). In addition, PBB-153 (43.40%), PBDE-153 (24.75%), and PBDE-85 (19.51%) were the most weighted in the WQS model (Figure 2A). Figure 2B shows a box diagram representing the weight (%) distribution for each BFR in the WQS model. PBB153 has the highest mass distribution and widest range, while other compounds have a smaller and more concentrated mass distribution. The figure illustrates the uncertainty and variability of the weights assigned to each compound in different models (Figure 2B).

**Table 2:** Univariate and multivariate analyses by the weighted linear model.

Exposure	Crude model			Model 1			Model 2		
	OR	95%CI	*p*	OR	95%CI	*p*	OR	95%CI	*p*
PBB153									
Continuous	1.32	1.09, 1.61	0.01	1.31	1.05, 1.64	0.02	1.23	0.96, 1.57	0.01
Q1	ref			ref			ref		
Q2	0.56	0.15, 2.02	0.37	0.52	0.14, 1.96	0.33	0.50	0.13, 1.89	0.30
Q3	5.58	2.19, 14.22	<0.001	5.40	2.02, 14.45	0.001	4.98	1.79, 13.86	0.003
Q4	3.46	1.18, 10.11	0.02	3.52	1.15, 10.70	0.03	3.23	1.03, 10.08	0.04
*p* for trend	<0.001			<0.001			0.001		
PBDE28									
Continuous	0.85	0.48, 1.50	0.57	0.86	0.49, 1.52	0.60	0.75	0.43, 1.31	0.31
Q1	ref			ref			ref		
Q2	1.00	0.39, 2.58	1.00	0.97	0.38, 2.52	0.95	0.91	0.34, 2.42	0.85
Q3	0.93	0.41, 2.10	0.86	1.03	0.45, 2.35	0.95	0.93	0.40, 2.17	0.86
Q4	0.97	0.41, 2.32	0.95	1.01	0.43, 2.40	0.98	0.80	0.33, 1.95	0.62
*p* for trend	0.91			0.95			0.65		
PBDE47									
Continuous	0.72	0.40, 1.30	0.27	0.75	0.42, 1.36	0.34	0.69	0.39, 1.24	0.21
Q1	ref			ref			ref		
Q2	0.74	0.34, 1.62	0.45	0.74	0.34, 1.63	0.45	0.72	0.32, 1.62	0.42
Q2	0.72	0.32, 1.65	0.44	0.84	0.37, 1.92	0.68	0.81	0.36, 1.85	0.61
Q4	0.67	0.30, 1.51	0.33	0.72	0.32, 1.62	0.43	0.63	0.28, 1.40	0.25
*p* for trend	0.35			0.50			0.32		
PBDE85									
Continuous	0.75	0.49, 1.14	0.18	0.81	0.54, 1.20	0.29	0.77	0.52, 1.13	0.17
Q1	ref		ref	ref					
Q2	0.92	0.43, 1.95	0.82	0.94	0.44, 2.01	0.88	0.94	0.44, 2.01	0.86
Q3	0.98	0.44, 2.19	0.96	1.17	0.51, 2.67	0.71	1.09	0.48, 2.47	0.83
Q4	0.54	0.22, 1.30	0.17	0.61	0.26, 1.45	0.26	0.55	0.23, 1.33	0.18
*p* for trend	0.26			0.48			0.33		
PBDE99									
Continuous	0.77	0.46, 1.29	0.31	0.81	0.49, 1.34	0.41	0.76	0.46, 1.26	0.27
Q1	ref			ref			ref		
Q2	0.86	0.39, 1.88	0.71	0.87	0.40, 1.90	0.73	0.87	0.39, 1.92	0.72
Q3	0.62	0.28, 1.35	0.22	0.70	0.32, 1.50	0.35	0.64	0.30, 1.36	0.24
Q4	0.76	0.30, 1.91	0.55	0.86	0.34, 2.18	0.75	0.76	0.30, 1.93	0.55
*p* for trend	0.42			0.61			0.43		
PBDE100									
Continuous	0.67	0.38, 1.18	0.17	0.70	0.40, 1.21	0.20	0.65	0.39, 1.11	0.11
Q1	ref			ref			ref		
Q2	1.52	0.65, 3.55	0.33	1.58	0.67, 3.71	0.29	1.52	0.65, 3.58	0.33
Q3	0.71	0.29, 1.73	0.44	0.80	0.33, 1.93	0.61	0.70	0.30, 1.64	0.40
Q4	0.69	0.27, 1.79	0.44	0.75	0.29, 1.94	0.55	0.68	0.27, 1.73	0.41
*p* for trend	0.21			0.31			0.17		
PBDE153									
Continuous	1.24	0.75, 2.05	0.39	1.25	0.76, 2.07	0.37	1.22	0.74, 2.01	0.42
Q1	ref			ref			ref		
Q2	0.78	0.28, 2.18	0.63	0.79	0.28, 2.24	0.66	0.79	0.29, 2.19	0.65
Q3	1.14	0.51, 2.53	0.75	1.05	0.45, 2.42	0.92	1.07	0.46, 2.48	0.87
Q4	1.25	0.48, 3.25	0.65	1.25	0.47, 3.33	0.64	1.19	0.45, 3.15	0.71
*p* for trend	0.47			0.51			0.56		
PBDE154									
Continuous	0.68	0.41, 1.14	0.14	0.72	0.44, 1.18	0.19	0.69	0.42, 1.11	0.12
Q1	ref			ref			ref		
Q2	1.07	0.50, 2.28	0.85	1.10	0.52, 2.35	0.80	1.09	0.51, 2.32	0.82
Q3	0.81	0.32, 2.01	0.64	0.91	0.37, 2.24	0.83	0.83	0.34, 2.04	0.68
Q4	0.71	0.29, 1.73	0.45	0.78	0.32, 1.88	0.57	0.71	0.30, 1.69	0.43
*p* for trend	0.39			0.54			0.38		
PBDE183									
Continuous	0.57	0.26, 1.25	0.15	0.83	0.43, 1.60	0.56	0.81	0.41, 1.59	0.53
Q1	ref			ref			ref		
Q2	0.64	0.26, 1.54	0.31	0.74	0.31, 1.76	0.49	0.76	0.32, 1.82	0.53
Q3	0.62	0.32, 1.21	0.16	0.89	0.46, 1.74	0.74	0.91	0.47, 1.78	0.78
Q4	0.66	0.28, 1.55	0.33	1.05	0.44, 2.50	0.91	0.99	0.40, 2.46	0.99
*p* for trend	0.26			0.96			0.97		
PBDE209									
Continuous	1.39	1.25, 1.56	<0.0001	1.50	1.33, 1.70	<0.0001	1.45	1.28, 1.64	<0.0001
Q1	ref			ref			ref		
Q2	1.24	0.63, 2.42	0.53	1.41	0.73, 2.69	0.30	1.39	0.72, 2.68	0.31
Q3	0.29	0.09, 0.96	0.04	0.37	0.11, 1.25	0.11	0.36	0.11, 1.22	0.10
Q4	0.57	0.25, 1.30	0.18	0.83	0.38, 1.82	0.63	0.77	0.35, 1.73	0.52
*p* for trend	0.03			0.20			0.16		

Crude model: no covariates were adjusted.

Model 1: adjusted for gender and race.

Model 2: adjusted for gender, age, race, poverty: income ratio, education level, marital status, BMI, smoking, and alcohol.

### Associations of all BFRs with PD in BKMR analyses

**Figure 3:**
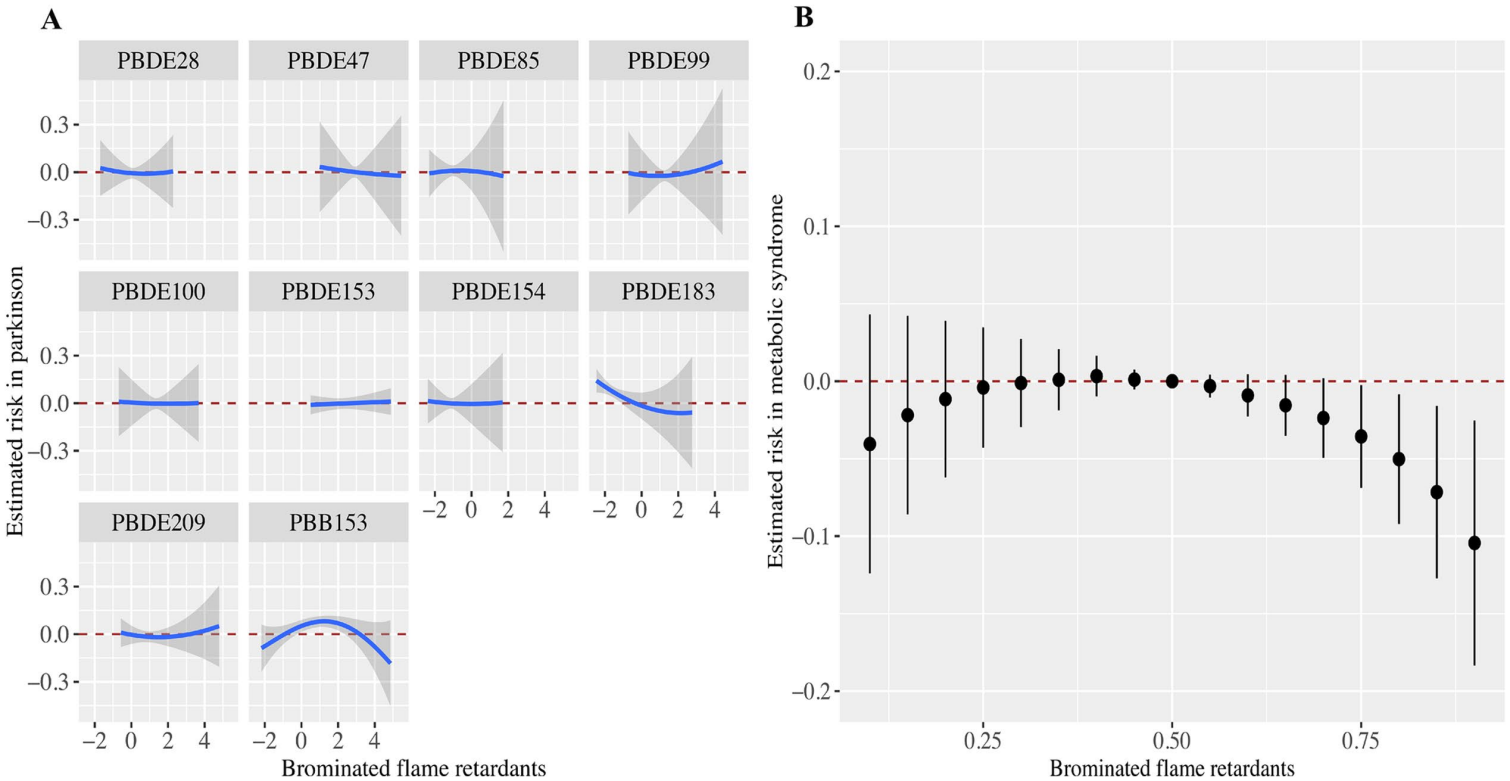
Associations of the serum brominated flame retardants (BFRs) content with PD risk estimated by Bayesian Kernel Machine Regression (BKMR). (A) Exposure-response functions for each BFRs with PD. (B) Combined effects of serum brominated flame retardants on PD risk. This plot showed the estimated difference in PD risk and 95% confidence interval when all BFRs concentrations were held at particular percentiles compared to their medians. Models were adjusted for gender, age, race, education, PIR, marital status, BMI, MET, drinking alcohol status, smoking status, and CCI.

We showed the association between serum BFRs and the risk of PD by BKMR model analysis (Figure 3). Interestingly, we found that most of the single BFRs were positively associated with PD, but PBB-153 showed an “inverted U” trend of first increasing and then decreasing the risk of PD. This is also in line with the findings of the above analysis, that is, the risk of PBB-153Q3 is 4.98 times that of Q1 (OR = 4.98, 95%CI: 1.79, 13.86, *p* < 0.05), and the risk of PBB-153Q4 is 3.23 times that of Q1 (OR = 3.23, 95%CI: 1.03 vs. 10.08, *p* < 0.05) (Table 2; Figure 3A). The risk of mixed BFRs and PD also reflected a trend that was similar to that seen in PBB-153, in which the risk first increased and then decreased (Figure 3B). In Figure 3B, we present the combined effects of all serum BFRs on PD risk. It is important to clarify that PBB-153 was included in the analysis alongside other BFRs. The estimated risk shown in the figure reflects the cumulative effect of all BFRs, including PBB-153, as their concentrations vary across different percentiles. This approach allows for a comprehensive assessment of the overall impact of BFR exposure on PD risk. In conclusion, serum BFRs showed an “inverted U” trend with PD.

### Nonlinearity analysis using RCS

**Figure 4:**
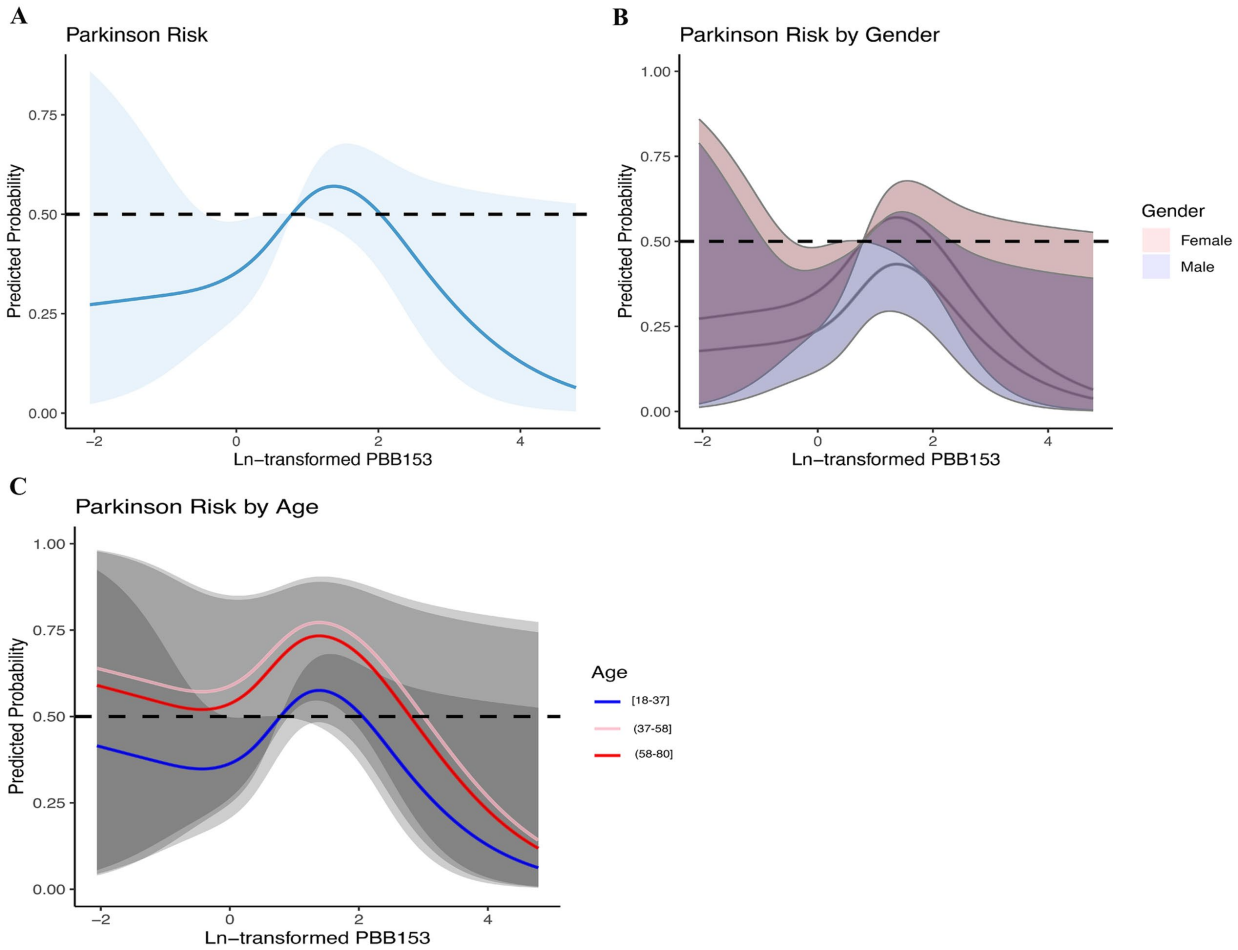
Relationship between brominated flame retardants and PD during 2009–2016. (A) Entirety; (B) By gender; (C) By age. Adjusted for age, sex, race, education, marital status, income, BMI, smoking, alcohol consumption, and CCI. The shaded area represents the 95% confidence interval.

Based on the above analysis, we suspected a nonlinear relationship between serum PBB-153 and PD incidence. To clarify the relationship between the two, we carefully examined the nonlinear relationship between PBB-153 concentration and the probability of developing PD after Ln transformation using penalized spline smooth curve fitting (Figure 4). After adjusting for potential confounders such as age, sex, race, education level, marital status, family income, body mass index, smoking status, alcohol consumption, and comorbidity index, the results of restricted spline analysis showed that the overall probability of developing PD first increased and then decreased with increasing serum PBB-153 concentration (Figure 4A), consistent with the findings of the above study. In addition, we found that the inverted U-shaped relationship between PBB-153 and PD was more pronounced in women and in people aged 37–58 years (Figures 4A,B).

### Subgroup analysis and sensitivity analysis

The results of subgroup analyses are shown in Table 3. Subgroup analysis showed that the *p* value for interaction between subgroups was >0.05, suggesting no significant effect change. In conclusion, the effect of serum PBB-153 on PD risk remained consistent across subgroups including sex, age, race, PIR, BMI, drinking status, and smoking status.

**Table 3:** Subgroup analyses on the association among brominated flame retardants with Parkinson.

Subgroup Variable	Bone mineral density		
	OR (95%CI)	*p*	*p-*Interaction
**Age**			0.121
[18, 37]	1.554 (0.850, 2.841)	0.15	
(37, 58]	1.379 (0.933, 2.038)	0.105	
(58, 80]	0.494 (0.334, 0.729)	<0.001	
**Gender**			0.119
Male	1.556 (1.231, 1.967)	<0.001	
Female	1.135 (0.796, 1.620)	0.478	
**Education**			0.391
Above high school	1.441 (1.062, 1.955)	0.02	
Under high school	1.176 (0.900, 1.536)	0.231	
High school or equivalent	1.168 (0.915, 1.492)	0.208	
**Marital status**			0.028
Widowed/divorced/separated	1.425 (1.052, 1.932)	0.023	
Married/cohabiting	0.918 (0.732, 1.150)	0.451	
Never married	1.403 (0.916, 2.149)	0.117	
**BMI**			0.773
[14.1, 18.5)	1.683 (1.283, 2.209)	<0.001	
[18.5, 28]	1.332 (1.013, 1.751)	0.04	
(28, 81.3]	1.280 (0.969, 1.693)	0.082	
**Poverty**			0.106
[0, 1.3)	1.371 (1.014, 1.855)	0.041	
[1.3, 3.5]	1.661 (1.248, 2.210)	<0.001	
(3.5, 5]	0.943 (0.572, 1.556)	0.816	
**MET**			0.427
[40, 600)	1.122 (0.878, 1.434)	0.351	
[600, 3, 000]	1.471 (1.173, 1.845)	0.001	
(3, 000, 47, 760]	1.130 (0.510, 2.503)	0.759	
**Smoke**			0.036
Former	0.926 (0.716, 1.198)	0.555	
Never	1.536 (1.083, 2.177)	0.017	
Now	1.278 (0.966, 1.690)	0.085	
**Alcohol**			0.07
Mild	1.214 (0.891, 1.654)	0.215	
Moderate	1.651 (1.318, 2.069)	<0.0001	
Former	1.008 (0.696, 1.459)	0.968	
Heavy	1.726 (1.360, 2.191)	<0.0001	
Never	1.010 (0.679, 1.503)	0.959	

OR, odds ratio; CI, confidence interval. The model is adjusted for age, gender, race, marital status, BMI, PIR, and educational level.

**Figure 5:**
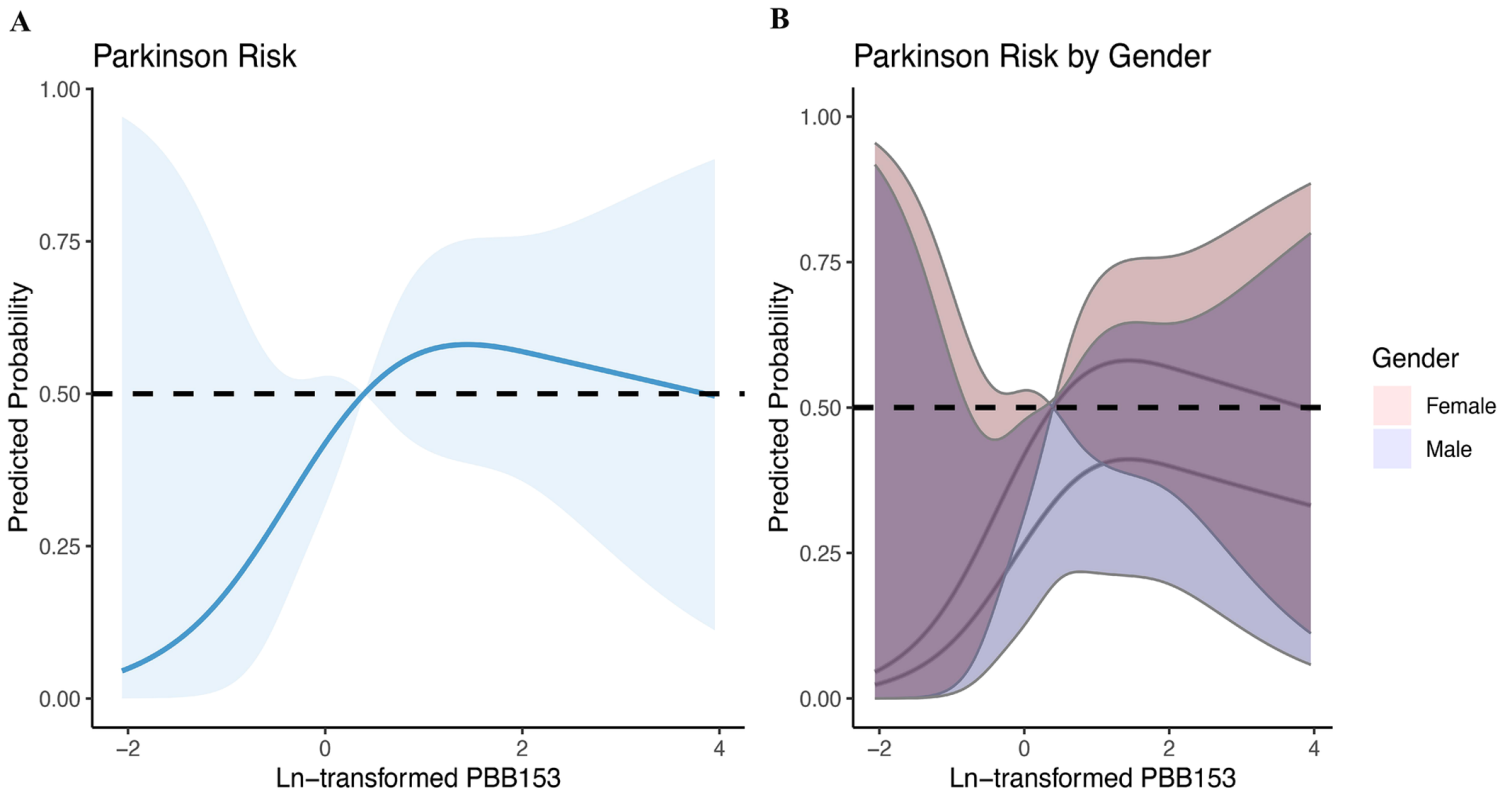
Sensitivity analysis—relationship between brominated flame retardants and PD during 2009–2016. (A) Entirety; (B) By gender. Adjusted for age, sex, race, education, marital status, income, BMI, smoking, alcohol consumption, and CCI. The shaded area represents the 95% confidence interval.

In addition, considering that increasing age is the most dominant risk factor for PD and may introduce susceptibility bias, we considered excluding the 60 years and older and critical population (*n* = 2,090) for sensitivity analysis to assess the robustness of the study. A total of 5,071 subjects were included in the sensitivity analysis. In the multivariate Logistic regression analysis, serum PBB-153 concentration was transformed into Ln to fit the normal distribution, and PBB-153 was divided into quartiles (Q1–Q4) to explore the relationship between different concentrations of PBB-153 and the risk of PD. The results showed that in model 2, for every unit increase in PBB-153 concentration, the prevalence of PD increased by 67% (OR: 1.67, 95%CI: 1.22, 2.27, *p* < 0.05). The risk of PBB-153 Q4 was 8.14 times higher than that of Q1 (OR = 8.14, 95%CI: 1.55, 42.85, *p* < 0.05) (Table 4). In addition, RCS analysis was performed on the subjects in the sensitivity analysis to further clarify the nonlinear relationship between PBB-153 concentration and the probability of developing PD after Ln transformation (Figure 5). The results showed an inverted U-shaped relationship between PBB-153 and PD (Figure 5A). Similar to these findings, we found that the inverted U-shaped relationship was more pronounced among women when we grouped according to sex (Figure 5B). In summary, the sensitivity results were similar to those described above, further validating the robustness of this study.

**Table 4:** Association between brominated flame retardants and Parkinson for sensitivity analysis.

Exposure	Crude model			Model 1			Model 2		
	OR	95%CI	*p*	OR	95%CI	*p*	OR	95%CI	*p*
PBB153									
Continuous	1.70	1.29, 2.23	<0.001	1.75	1.29, 2.36	<0.001	1.67	1.22, 2.27	0.002
Q1	ref			ref			ref		
Q2	2.18	0.43, 10.99	0.34	2.34	0.41, 13.31	0.33	2.26	0.39, 12.93	0.35
Q3	5.24	1.01, 27.07	0.05	5.01	0.87, 28.76	0.07	4.64	0.80, 26.99	0.09
Q4	7.81	1.82, 33.48	0.01	8.92	1.71, 46.51	0.01	8.14	1.55, 42.85	0.01
*p* for trend	0.003			0.003			0.01		

Crude model: no covariates were adjusted.

Model 1: adjusted for gender and race.

Model 2: adjusted for gender, age, race, poverty: income ratio, education level, marital status, BMI, smoking, and alcohol.

## Discussion

In this cross-sectional study, we utilized data from the nationally representative NHANES survey, covering the period from 2009 to 2016, to examine the potential association between exposure to various BFRs and the risk of PD among U.S. adults. Our findings reveal a non-linear, inverted U-shaped relationship between serum levels of PBB-153 and the likelihood of PD, even after adjusting for potential confounders. Specifically, PD risk increased with rising PBB-153 levels up to a threshold, beyond which the risk began to decline.

This non-linear association between PBB-153 exposure and PD risk is consistent with previous studies that have reported complex dose–response relationships between environmental exposures and neurodegenerative diseases (13–15). Several mechanisms may underlie this inverted U-shaped relationship. Initially, low levels of PBB-153 exposure could induce oxidative stress and neuroinflammation, both of which are well-established contributors to PD pathogenesis (16, 17). Oxidative stress leads to the accumulation of reactive oxygen species (ROS), which can damage cellular components such as proteins, lipids, and nucleic acids (18). Concurrently, neuroinflammation, marked by microglial activation and the release of pro-inflammatory cytokines, may exacerbate neuronal damage and contribute to neurodegeneration (19). These processes could potentially initiate or accelerate the progression of PD.

Conversely, at higher levels of exposure, PBB-153 may activate adaptive or protective mechanisms that reduce PD risk. One possible mechanism involves the activation of the nuclear factor erythroid 2-related factor 2 (Nrf2) pathway, which regulates the expression of antioxidant and detoxification enzymes (20). Exposure to certain environmental pollutants, including some BFRs, has been shown to induce Nrf2 activation, leading to the upregulation of cytoprotective genes and enhanced cellular defenses against oxidative stress (21, 22). This adaptive response might mitigate the neurotoxic effects of PBB-153 at higher exposure levels, resulting in a lower PD risk.

Another plausible explanation for the observed inverted U-shaped relationship is the phenomenon of hormesis, where low doses of a stressor elicit beneficial or adaptive responses, while higher doses become harmful (23). Hormetic effects have been proposed as a contributing factor in the pathogenesis of neurodegenerative diseases, including PD (24). At low levels of PBB-153 exposure, the mild cellular stress induced may activate adaptive pathways that enhance neuronal resilience, potentially preventing the accumulation of misfolded proteins, a hallmark of PD pathology (25). However, when exposure levels increase, the resultant cellular stress may exceed the capacity of these adaptive mechanisms, leading to neuronal dysfunction and cell death. The potential influence of age on this non-linear relationship also warrants consideration. Age is a well-known risk factor for PD, and our sensitivity analysis, which focused on participants younger than 60 years, revealed a more pronounced inverted U-shaped association between PBB-153 exposure and PD risk. This suggests that the non-linear relationship might be more prominent in younger individuals, possibly due to age-related differences in toxicokinetics, metabolic capacity, or cellular defense mechanisms (26).

The findings of this study are consistent with and build upon previous research exploring the link between environmental toxicants, such as BFRs, and PD. Earlier studies have suggested that certain BFRs, particularly PBDEs, may contribute to the development of neurodegenerative diseases by inducing oxidative stress and neuroinflammation (4, 6). Our study supports these mechanisms by demonstrating a significant association between BFR exposure and PD risk, specifically highlighting the inverted U-shaped dose–response relationship with PBB-153. This complex pattern aligns with previous work that has observed non-linear associations between environmental exposures and neurodegenerative outcomes, suggesting a hormetic effect where low-level exposure may initiate protective responses, but higher levels overwhelm these defenses, leading to neurotoxicity (23, 24).

Several potential biological mechanisms may explain the association between BFRs exposure and the risk of PD. One of the primary mechanisms involves oxidative stress, which is a well-established contributor to neurodegenerative diseases, including PD. BFRs, particularly PBDEs, have been found to increase the production of reactive oxygen species (ROS). ROS can lead to cellular damage, including the destruction of lipids, proteins, and nucleic acids, which subsequently results in the dysfunction and death of dopaminergic neurons, a key pathological feature of PD (6, 9, 18). Additionally, neuroinflammation is another significant mechanism. Exposure to BFRs may activate microglia, the immune cells in the central nervous system, leading to the release of pro-inflammatory cytokines. Chronic inflammation in the brain can exacerbate neuronal damage and accelerate the progression of neurodegenerative diseases such as PD (17, 19). Another possible pathway is mitochondrial dysfunction, which plays a central role in PD pathology. Studies suggest that BFRs can impair mitochondrial function, reducing the cells’ ability to produce energy and triggering apoptotic pathways, which can lead to the death of neurons in PD patients (16). Finally, the concept of hormesis, where low levels of exposure to toxicants like BFRs might initially activate protective mechanisms, but higher exposures lead to toxicity, may also explain the non-linear dose–response relationship observed between BFR exposure and PD risk. This biphasic response could involve adaptive cellular responses that temporarily mitigate neurotoxicity but ultimately fail at higher exposure levels (23–25). These mechanisms highlight the complex and multifactorial effects of BFRs on the central nervous system and underscore the need for further mechanistic studies to better understand the link between BFR exposure and PD.

In our study, we observed a more pronounced inverted U-shaped relationship between serum PBB-153 levels and Parkinson’s Disease (PD) risk in women compared to men. Several factors may contribute to this gender-specific effect. Firstly, women undergo significant hormonal changes throughout their lives, including during the menstrual cycle, pregnancy, and menopause (27). Estrogen, a key hormone, is known to influence neuroinflammatory responses and oxidative stress—two mechanisms critical in PD development (28). Estrogen’s modulation of neuroprotective pathways may alter the body’s response to environmental toxins like brominated flame retardants (BFRs), potentially heightening women’s vulnerability to neurotoxic effects of compounds such as PBB-153 (29).

Additionally, gender-specific differences in exposure patterns could play a role. Women are more likely to spend time in indoor environments where BFR-containing products, such as electronics and upholstered furniture, are common. This increased exposure through household dust and consumer products may result in higher internal doses of BFRs in women, thereby heightening the associated health risks, including the risk of developing PD (30). Moreover, physiological differences between men and women, such as body fat composition, liver enzyme activity, and kidney function, can affect BFR metabolism and elimination (31). These differences can lead to slower elimination rates and higher bioaccumulation of BFRs in women, resulting in prolonged exposure and potentially greater adverse effects on the nervous system (31, 32).

Our subgroup analysis further supported this, showing a more pronounced non-linear association between PBB-153 exposure and PD risk in women compared to men. This gender-specific difference may be attributed to a variety of factors, including hormonal influences, variations in exposure patterns, or differences in metabolic pathways and elimination rates (33, 34). This finding aligns with recent studies highlighting gender-specific effects of environmental exposures on neurodegenerative diseases. The influence of sex hormones, such as estrogen, which may provide neuroprotective effects, could explain this difference (33). Estrogen has been shown to modulate neuroinflammation and oxidative stress, both of which are critical mechanisms in PD pathogenesis (34). Further research is needed to explore the role of hormonal factors and whether postmenopausal women or those with lower estrogen levels are at higher risk when exposed to BFRs. In addition, previous research has documented gender-specific effects of environmental exposures on neurodegenerative diseases, underscoring the importance of considering potential effect modifiers in risk assessment (35, 36). What’s more, the results from the BMI subgroup analysis were also noteworthy. We observed that individuals with higher BMI showed stronger associations between BFR exposure and PD risk. This may be due to the fact that BFRs, being lipophilic compounds, are more likely to accumulate in adipose tissue. As a result, individuals with higher body fat may have prolonged and higher internal exposure to BFRs, leading to increased neurotoxic effects. This interaction suggests that obesity could act as a modifying factor, enhancing the adverse effects of BFR exposure on the nervous system. The WQS regression analysis further revealed that PBB-153, along with other BFRs such as PBDE-153 and PBDE-85, significantly contributed to the overall mixture effect on PD risk. This finding highlights the importance of considering the cumulative impact of multiple environmental exposures, rather than focusing solely on individual compounds (37). The combined effects of these BFRs may stem from their shared capacities to induce oxidative stress, disrupt endocrine signaling, or interfere with other cellular processes relevant to neurodegeneration (38, 39).

While this study provides valuable insights into the potential association between BFR exposure and PD risk, several limitations must be acknowledged. First, the cross-sectional design of the study precludes the establishment of causality, and the possibility of reverse causation cannot be entirely excluded. Longitudinal studies are necessary to better understand the temporal relationship between BFR exposure and the development of PD. Second, the identification of PD cases in the NHANES dataset relied solely on self-reported medication use, which may have led to misclassification of disease status. Incorporating more comprehensive diagnostic criteria, including clinical evaluations and neuroimaging data, would improve the accuracy of case identification. Third, the NHANES data did not capture information on potential confounding factors specific to PD risk, such as occupational exposures, history of head trauma, or genetic predispositions. Although adjustments were made for a range of sociodemographic and lifestyle factors, the potential influence of unmeasured confounders cannot be completely ruled out.

Despite these limitations, the present study has several strengths. The use of a nationally representative sample from the NHANES survey enhances the generalizability of the findings to the U.S. adult population. Additionally, the comprehensive assessment of multiple BFRs and the application of advanced statistical methods, such as WQS regression and BKMR, allowed for a more robust evaluation of the overall mixture effect and the identification of significant individual compounds. These methodological strengths contribute to a more nuanced understanding of the complex relationship between environmental exposures and PD risk.

In conclusion, this study provides evidence for a non-linear, inverted U-shaped relationship between serum levels of PBB-153 and the risk of Parkinson’s disease in U.S. adults. The findings highlight the importance of considering complex dose–response relationships and potential effect modifiers when assessing the impact of environmental exposures on neurodegenerative diseases. Further research, including prospective cohort studies and mechanistic investigations, is warranted to elucidate the underlying biological pathways and to inform public health strategies for mitigating the potential risks associated with BFR exposure.

## Conclusion

This nationally representative cross-sectional study identified a novel non-linear, inverted U-shaped relationship between serum levels of the brominated flame retardant PBB-153 and the risk of Parkinson’s disease in U.S. adults. The risk initially increased with rising PBB-153 exposure, only to decline beyond a certain threshold. This complex dose–response pattern underscores the importance of considering potential hormetic mechanisms and effect modifiers in the evaluation of environmental exposures and neurodegenerative diseases. Further research is needed to elucidate the underlying biological pathways and to inform the development of effective risk mitigation strategies.

## Data Availability

The original contributions presented in the study are included in the article/Supplementary material, further inquiries can be directed to the corresponding authors.
