# Supplementary material for: The association between brominated flame retardants exposure with Parkinson’s disease in US adults: a cross-sectional study of the National Health and Nutrition Examination Survey 2009–2016
Source: Front Public Health. 2024 Oct 21;12:1451686. doi: 10.3389/fpubh.2024.1451686 (PMC11532090; doi:10.3389/fpubh.2024.1451686)
Supplement: Supplementary file 1 [file Data_Sheet_1.docx]

**Supplementary materials**

Supplementary Figure 1. Pearson’s correlation matrix among brominated flame retardants in the study population.

Supplementary Figure 1. Pearson’s correlation matrix among brominated flame retardants in the study population.
